# Supplementary material for: The causal relationship between the human gut microbiota and pyogenic arthritis: a Mendelian randomization study
Source: Front Cell Infect Microbiol. 2024 Nov 26;14:1452480. doi: 10.3389/fcimb.2024.1452480 (PMC11629706; doi:10.3389/fcimb.2024.1452480)
Supplement: Supplementary file 1 [file DataSheet1.zip › mendelian test/ebi-a-GCST90027504.csv_leave_one_out.pdf]

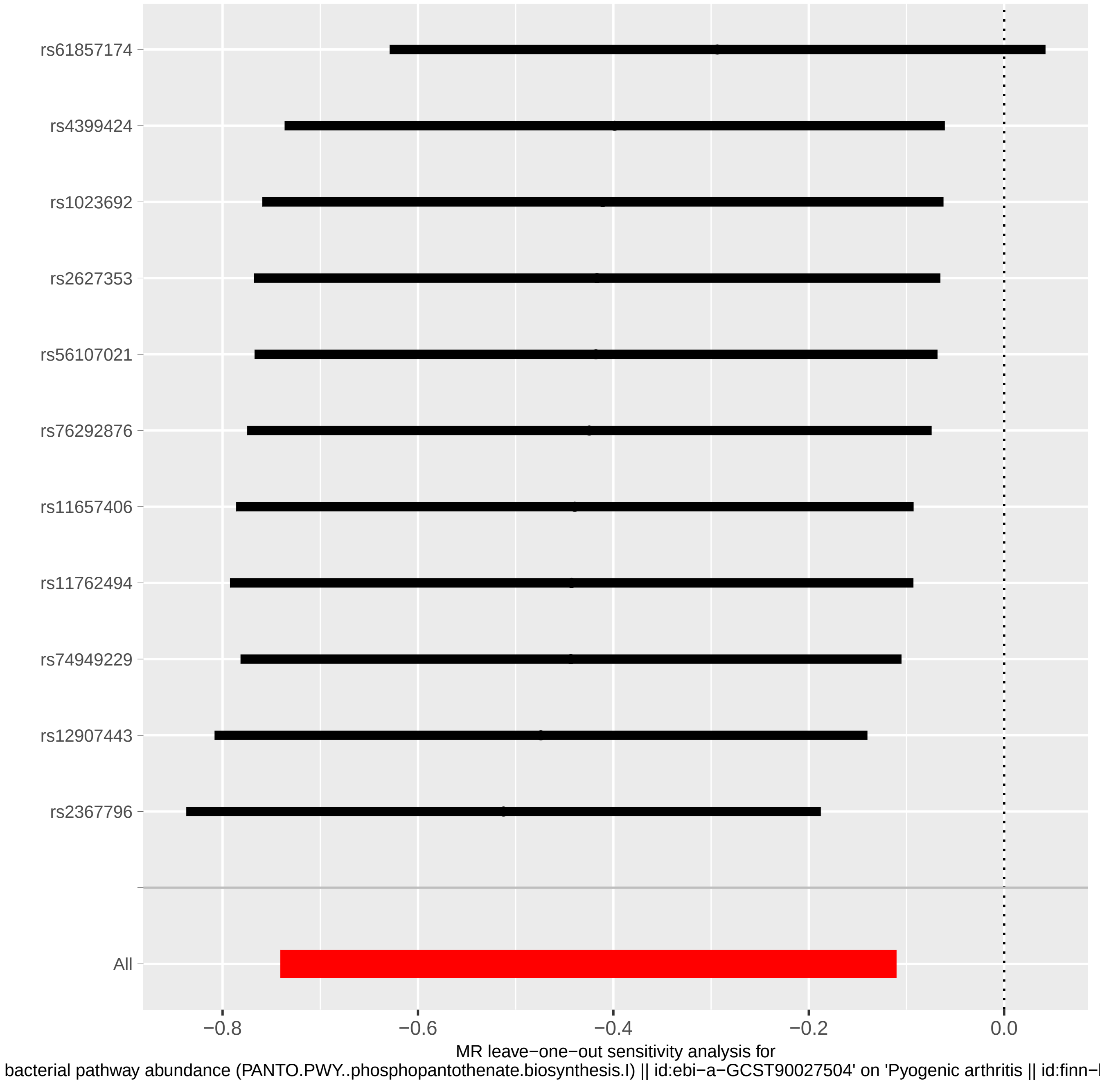
rs61857174
rs4399424
rs1023692
rs2627353
rs56107021
rs76292876
rs11657406
rs11762494
rs74949229
rs12907443
rs2367796
All
−0.8
−0.6
−0.4
−0.2
0.0
MR leave−one−out sensitivity analysis for
bacterial pathway abundance (PANTO.PWY..phosphopantothenate.biosynthesis.I) || id:ebi−a−GCST90027504' on 'Pyogenic arthritis || id:finn−
